# The Case for Fellowship Training in Emergency Psychiatry

**DOI:** 10.15694/mep.2020.000252.1

**Published:** 2020-11-11

**Authors:** Scott Simpson, Victoria Brooks, Dustin DeMoss, Ryan Lawrence

**Affiliations:** 1 Denver Health and Hospital Authority; University of Colorado Anschutz Medical Campus; 2 Erie County Medical Center; Jacobs School of Medicine and Biomedical Sciences; 3 JPS Health Network; University of North Texas Health Science Center of Fort Worth; 4 New York—Presbyterian Hospital; Columbia University Medical Center

**Keywords:** emergency psychiatry, behavioral emergencies, fellowship training, workforce development, crisis

## Abstract

This article was migrated. The article was marked as recommended.

Emergency departments (EDs) are a critical setting for behavioral health treatment particularly for minority and underserved communities, yet quality emergency psychiatric care remains inconsistently available. Subspecialty fellowship training in emergency psychiatry represents the most transformative potential approach to improving psychiatric care in EDs and crisis centers. We describe a new network of emergency psychiatry fellowship programs that are training a new generation of expert clinicians and leaders. Proposed educational milestones are described. These efforts will improve access to and the quality of mental health care for all patients regardless of treatment setting.

## Introduction

Every year over 10 million emergency department (ED) encounters in the United States involve a behavioral health concern ( Weiss *et al.*, 2016; Santillanes *et al.*, 2019). Compared to other ED patients, psychiatric patients experience longer lengths of stay, higher hospitalization and boarding rates, and greater mortality including of death by suicide ( Halmer *et al.*, 2015). Over 40% of persons who die by suicide are treated in an ED or urgent care in the year prior to death ( Ahmedani *et al.*, 2019). These numbers do not reflect the adverse influence of co-morbid mental illness on somatic conditions. Yet despite advances in understanding the epidemiology of behavioral emergencies and developing different models of care ( Halmer *et al.*, 2015), psychiatric patients remain undertreated in EDs and crisis clinics. These shortcomings particularly impact minority and underserved patients who disproportionately utilize EDs for care ( Kessler, Coates and Chanmugam, 2017).

The most transformative approach to improving the care of behavioral emergencies is developing fellowship programs dedicated to training physician-leaders in emergency psychiatry. The availability of emergency psychiatry fellowship training would advance the science, practice, and teaching of the subspecialty. In this editorial, we describe the need for these programs and introduce new fellowship programs that we are launching to meet this need.

## Current state of emergency psychiatry training

The United States’ Accreditation Council for Graduate Medical Education (ACGME) requires a structured emergency psychiatry experience for general psychiatry residents outside of on-call duties. However, the quality of these experiences varies, and many residents are expected to manage highly acute and complex psychiatric emergencies with minimal supervision ( Dennis and Swartz, 2015). There may be little commitment on the part of hospital-based services to improving behavioral health treatment in the ED, and residents may not learn the idiosyncrasies of psychiatric presentations and practice in the emergency setting. The resulting lower quality of emergency training is associated with higher burnout and decreased interest in treating safety net populations after residency ( Dennis and Swartz, 2015). Inadequate emergency psychiatry training thus impacts the accessibility of mental health care for all patients.

## The value of fellowship training

Experienced emergency psychiatrists generate different patient outcomes from less experienced colleagues. For instance, emergency psychiatrists admit fewer patients ( Moss *et al.*, 2018), and the patients they do admit require longer hospitalizations ( Hotzy *et al.*, 2018). These outcomes reflect the unique knowledge and perspective gained from expertise in emergency psychiatry and assertive use of alternative treatment models. The patient relationship may last only a single session, and the ability to work with patients may be impacted by the need to triage more acute presentations among a heavy workload, necessitating the development of sharp rapport-building and diagnostic skills. Patients are typically seen in states of crisis, including with intoxication, delirium, or undifferentiated illness requiring familiarity with acute medical and chemical dependency pathology. Patients with chronic illness may exhibit acute decompensation, in which there is an emphasis on recognition and pro-active mitigation of suicide and violence risk. Emergency psychiatrists are comfortable with this range of acuity for which inpatient psychiatric hospitalization ought to be considered a last resort. They leverage their familiarity with systems of care to avert hospitalization. These systems include local social, legal, and healthcare partners such as homeless shelters, police, and crisis lines. Better utilization of mental health services stands to realize cost savings for health systems without adversely impacting outcomes.

While the subspecialty of emergency psychiatry has matured, developing the field further requires a deeper commitment to fostering its future clinicians and leaders. Building emergency psychiatry fellowship programs would transform psychiatry’s approach to behavioral emergencies. For trainees, a clear pathway to practice in emergency psychiatry would make entering the field feel more inviting and plausible, particularly for residents with less robust emergency experiences. Faculty coalescing around fellowships would foster scholarship on priorities related to suicide prevention, the treatment of acute agitation and substance use disorders, social determinants of disease, and triage of psychotic symptoms in emergency settings ( Wilson *et al.*, 2019). Greater effort would focus on understanding outcomes of crisis care and adapting evidence-based psychotherapeutic and psychopharmacologic treatments to emergency practice. One example is honing the use of crisis intervention techniques to the constraints of a single ED visit ( Simpson, 2019). Fellowship programs are at once a response to the need for expertise in emergency psychiatry and also a prerequisite to advancing the field.

Better emergency psychiatry benefits mental health care more broadly. The availability of effective emergency behavioral health care is of concern to any community psychiatrist endeavoring to treat high risk patients or whose voicemail refers patients to a crisis line for emergencies. Readily available and effective emergency care enables community providers to feel more confident in managing sicker patients in less restrictive outpatient settings. Other clinical programming also depends on sound emergency psychiatric evaluations that drive community-based interventions; for example, sustaining intensive outpatient programs requires practitioners who can appropriately refer patients to those programs in lieu of acute hospitalization. In addition to these clinical benefits, the knowledge base and supervision processes developed for fellowships would benefit psychiatry residencies and other medical training programs. In this manner even trainees outside fellowships would gain greater confidence managing high-risk conditions including chronic suicidality and substance use disorders. Moreover, trainees would enjoy supervision from highly trained emergency psychiatry faculty.

These gains have not been realized by the current haphazard approach to emergency psychiatry. Hospitals use social workers, inpatient consult teams, telepsychiatry, emergency psychiatric evaluation services, or combinations thereof, to manage behavioral emergencies ( Halmer *et al.*, 2015). Some communities have additional levels of crisis care. The variety of practice settings has complicated defining standards of care, measuring quality of care, and recruiting psychiatrists into the field. Treating behavioral emergencies often falls to emergency physicians and social work clinicians who are not best trained for this work and consequently experience high rates of burnout. Fellowship programs would grow a community of psychiatrists well-suited and motivated to improve mental health care delivered in emergency settings. Finally, emergency psychiatry could subsume important topic areas currently outside the purview of extant subspecialties; these areas include paramedic and disaster psychiatry.

## A new fellowship experience

The authors have developed fellowship programs among 4 states designed to train expert clinicians and future leaders in the subspecialty of emergency psychiatry. Most clinical experiences are set in emergency and crisis behavioral health services. Additional core experiences include clinical rotations in pre-hospital settings with paramedics and police, and training in toxicology. Fellows are trained in patient triage and flow management, supervision of midlevel clinicians, and quantitative quality improvement methods-necessary skills for routinely managing larger numbers of acute patients. This content is not required by the current ACGME-accredited psychiatry fellowships, and inconsistently available among those fellowships that accommodate experiences in emergency settings. Table 1 describes a range of clinical scenarios that are common in emergency psychiatry but may not be encountered regularly in other fields. Our programs have developed a set of common educational milestones (Supplementary File 1) that may be used to guide curriculum development and support accreditation of the subspecialty.

**Table 1.** Emergency psychiatry topics related to existing subspecialties

Existing subspecialty	Related presentations in emergency psychiatry
Addiction psychiatry	Treatment of acute intoxication and incipient withdrawal syndromes, including managing associated behavioral disturbances and determining of level of care Identification and engagement of pre-contemplative patients with addiction who present for trauma or medical illness Identification of and intervention for sub-diagnostic use disorders (eg, at risk alcohol use) Management of Screening, Brief Intervention, and Referral for Treatment (SBIRT) teams at trauma centers
Child and adolescent psychiatry	Introduction of children and families to the mental health system during their first treatment episode Evaluation of acute suicidality and violent threats in the community and school settings, including collaboration with law enforcement regarding criminal behavior Rapid evaluation of parental adequacy affecting patient disposition Addiction management in children and adolescents Evaluation of children immediately after trauma
Consultation-liaison psychiatry	Evaluation of delirium and medical disposition in the pre-hospital setting Treatment of agitation in pre-hospital settings, and determining the most appropriate level of care (eg, inpatient medicine or psychiatry, nursing facilities, medical respite)Management and treatment of complex presentations including capacity for decision making in short time frames (minutes to hours rather than days)
Forensic psychiatry	Determination of whether charges should be pressed against patients in the aftermath of a violent episode, including regarding children Disposition determination to forensic or medical/psychiatric settings Support of medical staff injured by antisocial or aggressive patients Collaboration with law enforcement in community management of mental illness
Geriatric psychiatry	Evaluation for disposition to medical versus psychiatric units for treatment Management of community outreach and pre-hospital care Mitigation of delirium risk in the emergency department Disposition determination based on brief cognitive and functional assessments

This is an opportune moment for launching and sustaining emergency psychiatric fellowships. Patient demand for crisis care continues to grow, and jobs in the field for psychiatrists are plentiful. Fellowships will appeal to trainees seeking deeper training in the subspecialty or aspiring to leadership in the field. Increasing patient volumes enable financially viable clinical services to support fellowship programs, as at our institutions. This financial investment positions institutions to recruit psychiatrists who can safely, effectively, and efficiently manage emergency psychiatric services. Greater collaboration among professional societies in psychiatry and emergency medicine has raised the visibility of the field, offered networking opportunities, and supported standardization of training. The current environment differs from years past, when several isolated fellowships were developed and closed. Those programs suffered from inconsistent leadership and a subsequent lack of local institutional investment.

There are substantial hurdles to implementing successful programs and proving their worth. Extant fellowships in other fields could be adapted to enhance emergency experiences, thereby reducing the need for dedicated emergency psychiatry programs. There remain a variety of clinical providers and delivery models in crisis psychiatry; fellowships will need to demonstrate how highly credentialed graduates represent a worthwhile investment over less expensive midlevel providers or telepsychiatry contractors who are common in the field today. That the field lacks clearly defined quality care metrics and payment structures will only make it more difficult to make clear the value of subspecialty training.

## Conclusion

Patients experiencing psychiatric crisis deserve great care. Psychiatrists have too often failed to deliver. High quality mental health care must be available in EDs and crisis centers where patients present regardless of insurance or other co-morbidities that limit access to psychiatry treatment across other settings. Emergency psychiatry fellowship programs are the most promising approach to definitively improve how we treat patients with behavioral emergencies. Notwithstanding the challenges, the opportunity to build fellowships is here. We call for our colleagues to consider the potential of this subspecialty and support the development of these programs.

## Take Home Messages

• Over 10 million emergency department encounters a year in the United States are for behavioral health concerns, but quality emergency psychiatric care remains inconsistently available.
• New emergency psychiatry fellowship programs are being developed to train expert clinicians and prepare leaders in the subspecialty.
• These efforts will improve access to high quality mental health treatment for all patients regardless of treatment setting.

## Notes On Contributors

**Scott A. Simpson**, MD, MPH, is Medical Director of Psychiatric Emergency Services and director of the emergency psychiatry fellowship at Denver Health (CO). He is an Associate Professor of Psychaitry at the University of Colorado Anschutz Medical Campus. ORCID iD: https://orcid.org/0000-0002-4759-1595

**Victoria Brooks**, MD, is Medical Director of the Comprehensive Psychiatric Emergency Program and director of the emergency psychiatry fellowship at Erie County Medical Center and the Jacobs School of Medicine and Biomedical Sciences (NY).

**Dustin DeMoss**, DO, MS, is Medical Director of Trinity Springs Pavilion at JPS Health Network and Associate Professor of Psychiatry at the University of North Texas Health Sciences Center at Fort Worth. He is developing a new fellowship training program at that institution.

**Ryan E. Lawrence**, MD, is Medical Director of the Comprehensive Psychiatric Emergency Program at New York--Presbyterian Hospital and director of the emergency psychiatry fellowship at Columbia University Medical Center (NY). He is an Assistant Professor of Psychiatry at Columbia University Medical Center.
