# Supplementary material for: The Case for Fellowship Training in Emergency Psychiatry
Source: MedEdPublish (2016). 2020 Nov 11;9:252. [Version 1] doi: 10.15694/mep.2020.000252.1 (PMC10697437; doi:10.15694/mep.2020.000252.1)
Supplement: Peer review history

# Reviewer report: Reviewer response for version 1

**DOI:** 10.21956/mep.20191.r31237

**Published:** 2021-10-11

**Authors:** John Sandars

**Affiliations:** 1 Edge Hill University

This review has been migrated. The reviewer awarded 5 stars out of 5 The authors have provided a well justified personal view of the need for competence in emergency psychiatric care. Although the focus of the personal view is the US, there are important lessons for medical educators across the world.

Reviewer Expertise:

NA

No decision status is available

# Reviewer report: Reviewer response for version 1

**DOI:** 10.21956/mep.20191.r31235

**Published:** 2021-09-26

**Authors:** Julie Owen

This review has been migrated. The reviewer awarded 5 stars out of 5 These authors-- venerated experts in this important subspecialty of Psychiatry-- have constructed a thoughtful and well-supported case for formalized Emergency Psychiatry training. As a colleague working in this clinical niche, I cannot express strongly enough how valuable this piece is to any clinician attempting to improve or bolster emergency psychiatric care and education at his/her institution.

Reviewer Expertise:

NA

No decision status is available

# Reviewer report: Reviewer response for version 1

**DOI:** 10.21956/mep.20191.r31238

**Published:** 2021-07-28

**Authors:** Anna McDowell

This review has been migrated. The reviewer awarded 5 stars out of 5 This is a very enlightening article detailing the need for dedicated Emergency Psychiatry training and current efforts underway. The authors note challenges in the development of creating dedicated fellowships and note alternatives to include enhancing Emergency Psychiatry training in adjacent (ie Emergency Medicine, Psychosomatic Medicine, Addiction) fellowships and/or general residency programs. The demand for highly trained Emergency Psychiatry clinicians will only grow and this article outlines the reasons and steps to meet this need.

Reviewer Expertise:

NA

No decision status is available

# Reviewer report: Reviewer response for version 1

**DOI:** 10.21956/mep.20191.r31234

**Published:** 2021-04-09

**Authors:** Subha Ramani

**Affiliations:** 1 Harvard Medical School, Brigham and Women's Hospital

This review has been migrated. The reviewer awarded 5 stars out of 5 An excellent editorial written by experts in the field. The authors point out the important deficiencies in equitable healthcare for those with behavioural health disorders and emphasize the need for specialists especially in providing care for patients from underserved communities. I completely agree that high quality mental health care must be available in EDs and crisis centers where patients pfrom all walks of life can receive excellent care regardless of insurance or other co-morbidities. A general psychiatry training alone will not suffice to care for all the mental health needs of society in crisis situations and emergency psychiatry fellowship programs appear to be a promising approach to train clinicians to effectively manage patients with behavioral emergencies. As an internist who sees the extent of behavioural health disorders and their end results, I extend my appreciation to my Psychiatry colleagues to whom I often turn for advice.Our society needs their skills and expertise.All clinicians who see patients with mental health disorders, such as internists, paediatricians etc as well as psychiatrists should read this article.

Reviewer Expertise:

NA

No decision status is available

# Reviewer report: Reviewer response for version 1

**DOI:** 10.21956/mep.20191.r31236

**Published:** 2021-02-12

**Authors:** Nahed Khairy

This review has been migrated. The reviewer awarded 5 stars out of 5 At last, a comprehensive and courageous full article cheering Emergency Psychiatry as an independent subspecialty.

Reviewer Expertise:

NA

No decision status is available
